# Effects of Neuropilates on Functional Outcomes in Chronic Stroke: A Randomized Clinical Trial

**DOI:** 10.3390/healthcare12080850

**Published:** 2024-04-17

**Authors:** Cristina García-Bravo, Laura Delgado-Lobete, Rebeca Montes-Montes, Mª Pilar Rodríguez-Pérez, Nuria Trugeda-Pedrajo, Gemma Fernández-Gómez, Sara García-Bravo

**Affiliations:** 1 Department of Physical Therapy, Occupational Therapy, Physical Medicine and Rehabilitation, Research Group of Humanities and Qualitative Research in Health Science (Hum&QRinHS), Universidad Rey Juan Carlos, 28922 Alcorcón, Spain; cristina.bravo@urjc.es; 2 Department of Physical Therapy, Occupational Therapy, Physical Medicine and Rehabilitation, Research Group in Evaluation and Assessment of Capacity, Functionality and Disability (TO+IDI), Universidad Rey Juan Carlos, 28922 Alcorcón, Spain; pilar.rodriguez@urjc.es (M.P.R.-P.); nuria.trugeda@urjc.es (N.T.-P.); gemma.fernandez@urjc.es (G.F.-G.); 3 Departamento de Atención Sociosanitaria, Facultad de Ciencias Sociosanitarias, Universidad de Murcia, 30900 Lorca, Spain; rebeca.montes@um.es; 4 Department of Physical Therapy, Occupational Therapy, Physical Medicine and Rehabilitation, Universidad Rey Juan Carlos, 28922 Alcorcón, Spain; sara.garcia.bravo@urjc.es; 5 Physiocare Madrid, Physiotherapy Clinic, 28026 Madrid, Spain

**Keywords:** Neuropilates, stroke, occupational therapy, physiotherapy, Pilates, neurology

## Abstract

Neuropilates is an intervention approach that was developed as a modified version of the Pilates Method to be used for neurological rehabilitation. The main objective of this study was to analyze the effectiveness of regular physiotherapy and occupational therapy rehabilitation in comparison to a combination of traditional rehabilitation with Neuropilates in adults with post-stroke motor disabilities. This was a rater- and analyst-blinded randomized clinical trial with a three-month intervention and a one-month follow up. Participants were randomly allocated to either the experimental group (EG, receiving a combination of conventional therapy and Neuropilates; n = 15) or the control group (CG, receiving solely conventional therapy; n = 15). Once adjusted for baseline FIM scores, the results showed significant differences favoring the EG in daily functioning (FIM), static balance (FRT), right-hand manual dexterity (NHPT) and right-upper limb coordination (BBT). Satisfaction with the received treatment as measured with the CSQ-8 was significantly higher for the EG. In conclusion, the incorporation of Neuropilates, facilitated by a single experienced therapist, can be a valuable complement to conventional physical and occupational therapy. However, Neuropilates-based interventions should be supervised and tailored to each individual by a professional specifically trained in the method.

## 1. Introduction

The Pilates Method was created by Joseph Pilates in 1920 as a health-promotion approach aimed to help to control the position and movement of the body [1]. The Pilates Method consists of a series of body–mind exercises to enhance the stability, strength and flexibility of the body center or “core” by paying attention to muscle control, posture, and breathing [2]. It comprises 33 exercises based on seven principles: concentration, breathing, control, fluidity of movements, centralization and precision [1,2]. The Pilates Method has evolved from a simple exercise regimen to a recognized rehabilitation tool to reduce pain and disability, especially in patients with musculoskeletal and/or rheumatological conditions [3,4]. Specifically, within the field of neurological rehabilitation, Neuropilates was developed as a modified version of the Pilates Method that has been used to improve strength, postural control, alignment, stability, proprioception, balance, coordination and gait in people with neurological conditions [5,6].

Stroke survivors frequently face motor and cognitive deficits due to the brain damage, and movement disorders are prevalent clinical problems in this population [7,8]. In addition, challenges related to balance, mobility and/or strength are frequent and negatively impact survivors’ functional independence and performance, including an increased fall risk [4]. These difficulties frequently lead to limitations in the performance of daily living activities, so physical and occupational therapy are evidence-recommended interventions for this population to improve their daily performance and quality of life [7].

However, there is a lack of studies assessing the efficacy of Neuropilates for upper limb and daily performance outcomes in people who have experienced a stroke. Thus, this study aimed to analyze the effectiveness of regular physiotherapy and occupational therapy rehabilitation in comparison to a combination of traditional rehabilitation and Neuropilates in terms of static balance, upper limb coordination, gross and fine manual dexterity, and daily performance in adults with post-stroke motor disabilities.

## 2. Materials and Methods

### 2.1. Study Design

This was a 2-group randomized controlled trial (RCT) conducted in a rehabilitation center (Physiocare Madrid, Madrid, Spain), where both raters and the analyst were blinded to group allocation. This study included a 3-month intervention and a 1-month follow-up. Ethical approval was provided by the Universidad Rey Juan Carlos’s Research Ethics Committee (code: 3006202326123), and all participants were informed of the aims and methods of the study and provided written informed consent. The study followed the CONsolidated Standards of Reporting Trials (CONSORT) reporting guideline [9] and the protocol was registered in Clinical Trials (NCT06127485). In addition, the study followed the ethical principles of medical research involving human subjects in the Declaration of Helsinki, and it complied with national legislation, including Law 14/2007 on Biomedical Research and Royal Decree 223/2004.

### 2.2. Participants

Participants were recruited from the Physiocare Madrid rehabilitation center in Madrid. Inclusion criteria were: (1) aged 18 years or older; (2) had an ischemic or hemorrhagic stroke at least 6 months before study enrolment; (3) regularly attended physical or occupational interventions at Physiocare Madrid center; (4) a Barthel Index score of 65 or more; (5) a Mini Mental State Examination score of 18 or greater; and (6) not having a nasogastric tube. Exclusion criteria were other neurological conditions (i.e., tumors, anoxia, traumatic brain injury, neurodegenerative diseases, ataxia, aphasia, etc.), cardiorespiratory conditions, a Reisberg’s Global Deterioration Scale (GDS) stage of 6 or higher, which indicates a severe cognitive decline [10], a severely impaired level of consciousness or a Glasgow Coma Scale (GCS) score of 8 or lower, or receiving other complementary intervention therapy.

Patients who met the criteria were invited to participate in the study and were provided with a comprehensive information form detailing the potential risks and benefits of the trial and the procedures and interventions involved. Those who agreed to participate then signed the informed consent. Given the lack of studies regarding the effects of Pilates intervention on daily functioning in post-stroke patients, sample size calculations were carried out based on the findings regarding the effectiveness of a Pilates intervention on the quality of life of this population, as independent daily functioning is a main indicator of quality of life [2]. A sample size of n = 15 in each group was estimated to identify a previously reported effect size of 1.34 with a statistical power of >90% (α error < 0.05; β error < 0.90).

The randomization sequence was generated using the OxMaR program for minimization and randomization of clinical studies [11]. Participants were randomly allocated to (1) an experimental group (EG), receiving a combination of regular therapy and Neuropilates, or (2) a control group (CG) receiving regular physiotherapy and occupational therapy, with an allocation ratio of 1:1 (EG;CG). Each participant’s sequence number was delivered in an opaque envelope and provided to the investigators by a professional not involved in running the trial. Outcome variables were individually assessed by physical and occupational therapists blinded to participants’ allocation. Two occupational therapists and a physiotherapist, other than the one who conducted the sessions, performed all the assessments (baseline, end of trial and follow-up). Analyses were also performed by one investigator (L.D.-L.) blinded to participants’ allocation. Participants and the therapists who delivered the interventions were not blinded.

### 2.3. Intervention

**Figure 1:**
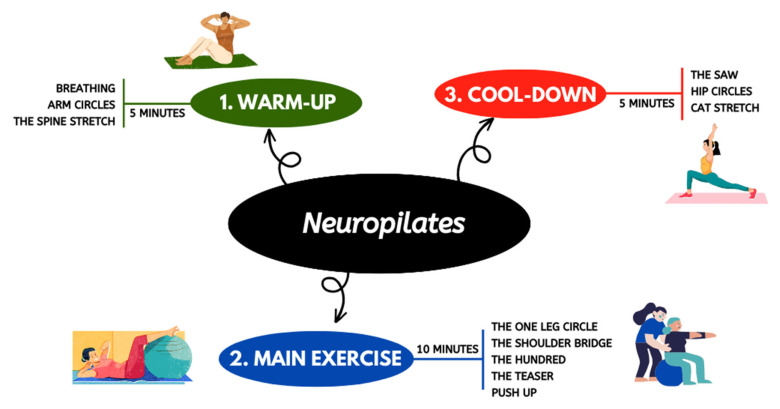
Pilates intervention protocol for EG.

Each intervention group attended twice-weekly intervention sessions of 60 min for 3 months at Physicare Madrid center. Participants in the CG received 30 min of regular physiotherapy followed by 30 min of regular occupational therapy. Regular physiotherapy included lower limb stretching techniques and muscle activation, and gait reeducation. Conventional occupational therapy included upper limb stretching techniques and muscle activation, and basic activities of daily living training (dressing, eating and swallowing, bathing, toileting and toilet hygiene). Participants in the EG received 20 min of regular physiotherapy, 20 min of conventional occupational therapy and 20 min of Neuropilates, which comprised activation warm-up exercises, main exercises, and cool-down exercises performed on a mat, stretcher or chair (Figure 1). The Neuropilates protocol was individually tailored to meet each participant’s difficulties or disabilities. In this protocol we worked with motor control exercises and strengthening of the upper and lower limbs, as well as balance and strengthening of the abdominal musculature (Supplementary Material S1). All Neuropilates sessions were performed by an occupational therapist with more than 9 years of experience in the therapeutic Pilates Method and Neuropilates.

### 2.4. Measures

Measures were assessed for both groups at baseline, three months (i.e., end of intervention) and four months (i.e., one-month follow-up) by blinded therapists.

#### 2.4.1. General Medical Information

Information regarding the overall health status of the participants was gathered using an ad hoc questionnaire that assessed participant sex, age, months since stroke, stroke type, medical and social background and current weekly hours of physical and occupational therapy intervention.

#### 2.4.2. Functional Outcomes

- Functional Independence Measure (FIM) [12] was used to assess functional independence of activities of daily living, and it was the main outcome. It comprises 18 items regarding motor and cognitive daily performance. The score ranges from 1 to 7, with a higher score indicating a higher level of functional independence. Administration was carried out through task observation (eating, grooming, bathing, dressing—upper body, dressing—lower body, toileting, transfers between bed, chair, and wheelchair, toilet, tub/shower, walk and stairs) and an interview (bladder management, bowel management, auditory comprehension, verbal expression, social memory). The minimal clinically important difference values for the FIM in patients post-stroke are 22, 17 and 3 for the total FIM, motor FIM and cognitive FIM, respectively [13].
- Functional Reach Test (FRT) [14] was used to measure the maximum distance an individual can move their gravity towards the limits of their support area (i.e., static balance). In a relaxed standing posture, with the feet at hip height, the person is instructed to do a shoulder flexion up to 90 degrees and to keep the hand extended. The person must go forward as much as they can, and the examiner then records the final distance in centimeters. Two preliminary practice trials were administered to each participant preceding the performance of three FRT trials. The final score was determined by calculating the mean score derived from the three trials.
- Timed ‘Up and Go’ (TUG) [15] was used to measure overall balance and gait speed. The person is asked to get up from a chair, walk 3 m, turn, return to the chair and sit down. The final score is recorded in seconds, with a higher score indicative of a diminished balance.

#### 2.4.3. Upper Limb Performance Outcomes

- Nine Hole Peg Test (NHPT) [16] was used to assess manual dexterity as it is considered a gold-standard test for this outcome. Each participant was asked to place nine pegs on a board with 9 holes and to remove them again, as quickly as possible. The final score is recorded in seconds, with a higher score being indicative of a diminished manual dexterity. Each hand was scored separately.
- Box and Block Test (BBT) [17] was used to assess overall upper limb gross motor performance. It is composed of a wooden box divided in two compartments by a partition, one of which contains 150 wooden blocks. Each participant was asked to move, one by one, as many wooden blocks as possible from one compartment to the other within 60 s. Scores were based on the number of blocks transferred, with higher scores being indicative of a better upper limb performance. Each arm was scored separately.
- Disabilities of the Arm, Shoulder, and Hand Questionnaire (DASH) [18] was used to assess daily activity and participation according to the 9 domains described in the International Classification of Upper Limb Functioning, Disability, and Health.

#### 2.4.4. Secondary Outcomes

The Client Satisfaction Questionnaire (CSQ-8) [19] was used to assess the level of satisfaction with the professional care and intervention received, as well as the degree of compliance with the patient’s expectations prior to the intervention. It is a self-administered questionnaire comprising eight questions ranging from 1 to 4, where higher values indicate a greater satisfaction. It has been validated in the Spanish population, showing adequate psychometric properties [20].

### 2.5. Statiscal Analysis

Sample size calculation was performed using G*Power for Windows, version 3.1.9.7 (Heinrich-Heine-Universitäta Düsseldorf, Düsseldorf, Germany). Statistical analyses were performed using IBM SPSS Statistics for Windows, version 25.0 (IBM SPSS Corp., Armonk, NY, USA). Data from the pre-, post- and follow up outcome measures were examined for normality using visual inspection and skewness and kurtosis, assuming that values >2 and >7 for skewness and kurtosis were indicative of normal distribution [21]. All data fall between these values except for post-intervention TUG (skewness = 2.1), follow-up BBT (right hand, skewness = 2.3; left hand, kurtosis = 8.4). As these values were near normality and only present for three variables, parametric tests were used. Descriptive and bivariate statistics were used to summarize and compare the sociodemographic, clinical and baseline functional characteristics of the participants across groups. In order to assess the aims of the study, between- and intra-group comparisons of primary and secondary outcomes at the end of the intervention and follow-up were performed using *t* tests. Finally, we performed an ANCOVA analysis of post-intervention and follow-up outcomes adjusted for baseline FIM scores. ANCOVA assumptions were examined and met for all dependent variables but for TUG (both post-intervention and follow-up scores). Accordingly, a bootstrapping adjustment for ANCOVA was performed for post-intervention and follow-up TUG. Statistical significance was set at *p* < 0.05 (two-sided).

## 3. Results

### 3.1. Attrition Rate

**Figure 2:**
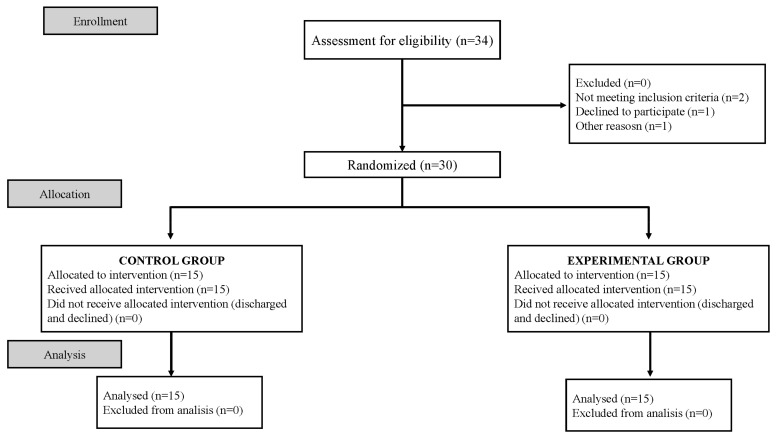
Flow diagram of the experimental procedure.

All 30 of the enrolled participants successfully completed the 1-month follow-up, resulting in a retention rate of 100%. A flow diagram of the experiment is shown in Figure 2.

### 3.2. Baseline Characteristics of Participants

The baseline sociodemographic, clinical, and functional characteristics of the 30 participants are reported in Table 1. Both groups were significantly similar regarding age, sex, months since stroke, type of stroke, hand dominance, impaired upper limb and weekly hours of physical therapy intervention, although participants from the experimental group received fewer weekly hours of occupational therapy than participants form the control group (CG = 1.9 [0.3] vs. EG = 1.6 [0.5]; *p* < 0.05). Participants were mostly men (56.6%) with a mean age of 57.6 (14.0) years and were predominantly right-handed (83.3%). A hemorrhagic stroke was diagnosed for most participants (73.3%), and months since stroke ranged from 13 to 121 months, with an overall mean of 42.3 (27.5).

Regarding their baseline functional status, both groups were statistical similar except for in terms of their general performance as measured with the FIM (CG = 68.0 [6.4] vs. 74.5 [7.7]; *p* < 0.05) and right-hand manipulation as measured with the NHPT (111.9 [48.1] vs. 77.0 [31.7]; *p* < 0.05), where the experimental group showed better baseline functioning.

### 3.3. Effects of the Intervention

#### Primary Outcomes

Regarding intra-group differences (Table 2), the participants in the EG had significantly improved all outcomes from baseline to the end of intervention, and the improvements were maintained during the follow-up for all variables but BBT (both right and left hands). Conversely, the intervention for the CG only yielded significant differences for FIM, NHPT (right hand) and BBT (left hand), for both post-intervention and follow-up outcomes. However, neither group achieved the minimal clinically important difference in the FIM overall score.

The results showed that were significant group differences in favor of the experimental group in the FIM, the FRT, the NHPT (right hand), the BBT (right hand) and the DASH (occupation score; Table 2). There were no significant inter-group differences on the TUG, the NHPG (left hand), the BBT (left hand) and the DASH (disability score). Furthermore, changes gained following the intervention remained after the follow-up month for the FIM, the FRT, the NHPT (right hand) and the DASH (occupation score), but not for the BBT (right hand; Table 3).

In addition, findings from the ANCOVA, once adjusted for baseline FIM scores, showed that, while post-intervention and follow-up FIM, FRT and NHPT (right hand) outcomes were still significantly and moderately different according to intervention group allocation, the baseline FIM scores influenced the effectiveness of the intervention on the DASH disability and occupation scores and NHPT (left hand, Table 4).

In terms of secondary outcomes, satisfaction with received treatment as measured with the CSQ-8 was significantly higher for the experimental group (CG = 27.8 [3.1] vs. EG = 30.0 [1.9]; *p* = 0.029).

## 4. Discussion

To the best of our knowledge, this study is the first RCT to incorporate Neuropilates into regular physiotherapy and occupational therapy as an intervention for individuals recovering from a stroke. This study addressed this gap by implementing a combined intervention of regular physiotherapy, occupational therapy and Neuropilates, comparing it with a conventional intervention alone. Our findings indicate that, over a three-month period, the integration of Neuropilates significantly improved static balance, right-hand manual dexterity and the daily functioning of stroke survivors. In addition, adherence to treatment was excellent, as was perceived satisfaction with the applied protocol. Improvements after regular physiotherapy and occupational therapy have been demonstrated in people with upper and lower limb and balance issues following a stroke [22,23,24,25]. Mobilization and stretching techniques of the upper limb such as the Bobath concept or constraint-induced movement therapy have proven effective in the recovery of the upper limb [23,24]. However, the scientific evidence for the use of Neuropilates as an adjunct rehabilitation tool in this population is still limited [4,6].

Currently, there is no consensus on the application of standardized protocols of Neuropilates in people who have experienced strokes, largely due to the lack of scientific literature in this regard. Cronin and Monaghan [6] have contributed to this topic by publishing a protocol for a randomized controlled feasibility study tailored towards stroke survivors with more than six months of evolution, similar to the present study participants. However, their protocol was designed to be implemented online Neuropilates sessions conducted remotely, consisting of six weeks of three-weekly sessions of 1 h each, two of which were delivered without professional supervision. In contrast, our participants received a combination of 20 min of Neuropilates and 40 min of regular physiotherapy and occupational therapy per session, twice a week for three months. Moreover, all sessions were conducted face-to-face and were performed under professional supervision, which allowed for dynamic adjustments and tailored adaptations based on each participant’s strengths and difficulties. Other researchers have conducted Pilates sessions lasting between 30 and 60 min, occurring two or three times weekly, over durations ranging from 8 to 12 weeks [2,26,27].

Few additional studies have used Neuropilates as an intervention approach for stroke patients [26,27,28,29]. Yun et al. [26] concluded that performing a Pilates-based exercise routine involving several repetitions of varying intensities improved the physical capacity and quality of life of stroke survivors. In this line, Sung Lim et al. [27] found that this method can also enhance the cardiopulmonary function of this population which, in turn, contributes to their functional performance. More similarly to the present design, Shea and Moriello [28] reported that Pilates should be used in combination with conventional intervention approaches, as the combination produced improvements in balance, lower limb strength and overall quality of life. In addition, these authors highlighted that all interventions involving the Pilates Method or a Pilates-based approach should be performed under professional supervision [28]. A systematic review conducted by Walter et al. [2] concluded that employing the Pilates Method or a Pilates-based approach for stroke rehabilitation led to improvements in functional, dynamic and static balance while enhancing reaction time to stimuli and overall quality of life. Our findings align with theirs, demonstrating positive outcomes regarding static balance, manual dexterity and daily functional independence among people who have experienced a stroke.

The efficacy of the Pilates Method has been studied across several health conditions, regardless of the mode of application [26,27,28,29,30,31,32,33,34]. However, Park et al. [35] argued that the extent of the benefits is dependent on the mode of delivery, highlighting that this approach is more effective when provided in face-to-face sessions under the supervision of professionals trained in the method, in comparison to online or telerehabilitation delivery, as in-person intervention allows for a more precise exercise performance, and an expert instructor can tailor the exercises to meet the demands of the participants. Conversely, Walter et al. [2] pointed out that, when used for rehabilitation purposes, Pilates sessions should be conducted by professionals not only trained in the method but also possessing experience in the therapeutic intervention of individuals who experienced a stroke. In the present study, in-person sessions were provided by an occupational therapist certified in the Pilates Method, which not only ensured the precision of exercise execution but also made it possible to tailor adaptations and modifications to each exercise based on the participants’ individual characteristics.

Other authors have also investigated the efficacy of the Pilates Method in diverse neurological conditions such as Parkinson Disease [30], multiple sclerosis [31,32], long-COVID [33] or Stiff Person Syndrome [34], particularly focusing on its effects on lower limb functioning and quality of life [30,31,32,33,34]. For instance, Çoban et al. [30] conducted a study comparing Pilates with regular physiotherapy in people with Parkinson Disease, implemented twice a week over 8 weeks. Both groups showed improvements in lower limb strength, fall risk and functional mobility post-intervention, with the Pilates group demonstrating additional enhancements in dynamic balance. In the same line, Eldemir et al. [31] and Najafi et al. [32] reported positive outcomes in gait, balance, fatigue, strength, stability and quality of life for individuals with multiple sclerosis receiving face-to-face Pilates intervention or telerehabilitation. Belgen Kaygisiz et al. [34] also concluded that a face-to-face Pilates-based intervention, following an 8-week protocol, yielded physical benefits in terms of balance and gait for people with Stiff Person Syndrome. Our findings align with these studies regarding lower limb and static balance benefits and provide novel information regarding upper limb and daily performance in stroke survivors.

In addition, understanding patients’ healthcare and intervention experience is particularly important, as a positive experience is associated with greater satisfaction, adherence to treatment, compliance and intervention persistence. Therefore, using Patient-Reported Experience Measures (PREM) and Patient-Reported Outcome Measures (PROM) is essential and should be implemented in trials focusing on the effects of a therapeutic intervention [36,37,38]. In our study, the group undergoing combined regular therapy with Neuropilates reported significantly higher satisfaction in comparison to regular therapy alone. Thus, although it is not currently possible to establish a standardized, evidence-based protocol for the implementation of Neuropilates in stroke survivors, both as a stand-alone intervention and as an adjunctive intervention approach, these findings contribute to understand the features that such a protocol should include.

This trial has several limitations. First, the small sample should be increased in future studies to validate these findings. It would also be recommended that future research on this topic increases length of the Neuropilates intervention or its isolates it to compare it to conventional rehabilitation therapy. The difference in the baseline sociodemographic characteristics of the participants (number of hours of occupational therapy per week) may have had an impact on the results. Future studies should avoid baseline differences to test the efficacy of Neuropilates as a valid rehabilitation method. In addition, while intra- and inter-group differences in the main primary outcome (i.e., FIM) were statistically significant, the improvements were not clinically meaningful according to the determined cutoffs. This could be due to the small sample size, as the effect size findings suggest that the changes are of moderate to large effect. Additionally, only one rehabilitation center participated in the study, which may limit the generalization of the findings. Thus, it would be advised to further contrast these results in larger and multicentric samples. Last, given the short-term follow-up in our study, future studies should extend into medium and long-term follow-ups in order to analyze whether the effects of this combined approach persist for longer periods of time.

## 5. Conclusions

The implementation of Neuropilates, facilitated by a single experienced therapist, may be a valuable adjunct therapeutic tool to regular physiotherapy and occupational therapy in people who have experienced a stroke. This combination demonstrates significant improvements in static balance, upper limb coordination, manual dexterity and daily activities performance than physical and occupational therapy alone, and it leads to a higher patient satisfaction. However, Pilates-based interventions need to be supervised and individually tailored to the patients’ characteristics by professionals specifically trained in the method.

## Figures and Tables

**Table 1:** Baseline characteristics of participants (n = 30).

Characteristic	Control Group n = 15 Mean (SD)/N (%)	Experimental Group n = 15 Mean (SD)/N (%)	*p* Value
Age	55.7 (16.8)	58.5 (11.1)	0.732
Sex			0.713
Male	8 (53.3)	9 (60.0)	
Female	7 (47.7)	6 (40.0)	
Months since diagnosis	50.1 (33.4)	34.5 (18.0)	0.128
Stroke			0.409
Hemorrhagic	10 (66.7)	12 (80.0)	
Ischemic	5 (33.3)	3 (20.0)	
Hand dominance			0.624
Right	12 (80.0)	13 (86.7)	
Left	3 (20.0)	2 (13.3)	
Impaired upper limb			0.143
Right	9 (60.0)	5 (33.3)	
Left	6 (40.0)	10 (66.7)	
Occupational Therapy intervention weekly hours	1.9 (0.3)	1.6 (0.5)	0.034 *
Physical Therapy intervention weekly hours	1.7 (0.5)	1.8 (0.4)	0.679
FIM ^a^	68.0 (6.4)	74.5 (7.7)	0.018 *
FRT ^b^	2.6 (1.8)	2.7 (2.0)	0.847
TUG ^c^	110.3 (49.6)	93.4 (26.4)	0.254
NHPT ^d^ (right)	111.9 (48.1)	77.0 (31.7)	0.027 *
NHPT ^d^ (left)	90.1 (40.4)	96.2 (30.8)	0.645
BBT ^e^ (right)	29.9 (31.0)	51.3 (31.3)	0.068
BBT ^e^ (left)	41.6 (29.5)	28.7 (26.2)	0.214
DASH disability	85.3 (8.7)	86.1 (7.6)	0.790
DASH occupation	94.1 (7.5)	91.1 (8.7)	0.320

^a^ = Functional Independence Measure; ^b^ = Functional Reach Test; ^c^ = Timed Up and Go Test; ^d^ = Nine Hole Peg Test; ^e^ = Box and Block Test. * *p* < 0.05.

**Table 2:** Intra-group differences in outcomes from baseline to end of intervention and follow-up (functional outcomes; n = 30).

Experimental Group					
Outcomes	Pre-Intervention Score (M [SD])	Post-Intervention Score (M [SD])	*p* Value	Follow-Up Score (M [SD])	*p* Value
FIM ^a^	74.5 (7.7)	82.1 (6.7)	<0.001 *	82.3 (6.8)	<0.001 *
FRT ^b^	2.7 (2.0)	5.7 (2.6)	<0.001 *	5.5 (2.6)	<0.001 *
TUG ^c^	93.4 (26.4)	91.0 (26.4)	0.008 *	87.3 (24.2)	<0.001 *
NHPT ^d^ (right)	77.0 (31.7)	67.5 (20.9)	0.007 *	63.2 (18.6)	0.002 *
NHPT ^d^ (left)	96.2 (30.8)	82.3 (21.7)	<0.001 *	80.8 (22.4)	<0.001 *
BBT ^e^ (right)	51.5 (31.3)	56.4 (31.3)	<0.001 *	58.6 (32.6)	0.427
BBT ^e^ (left)	28.7 (26.2)	35.7 (27.1)	<0.001 *	50.1 (54.9)	0.134
DASH disability	86.1 (7.6)	82.3 (7.9)	<0.001 *	81.7 (7.6)	<0.001 *
DASH occupation	91.1 (8.7)	86.1 (8.5)	<0.001 *	85.5 (9.1)	<0.001 *
Control group					
Outcomes	Pre-intervention Score (M [SD])	Post-intervention score (M [SD])	*p* Value	Follow-up score (M [SD])	*p* Value
FIM ^a^	68.0 (6.4)	70.2 (6.5)	0.002 *	70.5 (6.5)	0.002 *
FRT ^b^	2.6 (1.8)	2.8 (1.7)	0.098	2.6 (1.8)	0.974
TUG ^c^	110.3 (49.6)	108.6 (47.8)	0.080	106.1 (49.6)	0.064
NHPT ^d^ (right)	111.9 (48.1)	109.4 (47.3)	<0.001 *	109.1 (47.5)	0.007 *
NHPT ^d^ (left)	90.1 (40.4)	89.5 (41.4)	0.173	89.0 (42.3)	0.289
BBT ^e^ (right)	29.9 (31.0)	30.8 (31.6)	0.097	35.2 (32.8)	0.583
BBT ^e^ (left)	41.6 (29.5)	42.7 (30.1)	0.048 *	43.0 (30.6)	0.022 *
DASH disability	85.3 (8.7)	84.5 (8.6)	0.138	84.5 (8.7)	0.200
DASH occupation	94.1 (7.5)	93.1 (7.2)	0.161	93.0 (7.3)	0.136

^a^ = Functional Independence Measure; ^b^ = Functional Reach Test; ^c^ = Timed Up and Go Test; ^d^ = Nine Hole Peg Test; ^e^ = Box and Block Test. * *p* < 0.05.

**Table 3:** Inter-group differences in outcomes from baseline to end of intervention and follow-up (EG vs. CG; functional outcomes; n = 30).

	Post-Intervention			Follow-Up		
Outcomes	Inter-Group Difference	[95% CI]	*p* Value	Inter-Group Difference	[95% CI]	*p* Value
FIM ^a^	11.9	[7.0, 16.9]	<0.001 *	11.7	[6.8, 16.7]	<0.001 *
FRT ^b^	2.9	[1.2, 4.5]	0.001 *	2.9	[1.2, 4.6]	0.001 *
TUG ^c^	−17.6	[−46.4, 11.3]	0.222	−18.9	[−48.1, 10.3]	0.196
NHPT ^d^ (right)	−41.9	[−69.8, −13.9]	0.005 *	−45.9	[−73.6, −18.2]	0.003 *
NHPT ^d^ (left)	−7.2	[−32.3, 17.9]	0.557	−8.2	[−33.9, 17.5]	0.513
BBT ^e^ (right)	25.6	[2.1, 49.1]	0.034 *	23.4	[−1.1, 47.9]	0.060
BBT ^e^ (left)	−7.0	[−28.4, 14.4]	0.509	7.1	[−26.2, 40.3]	0.667
DASH disability	−2.2	[−8.4, 4.0]	0.471	−2.8	[−8.9, 3.3]	0.355
DASH occupation	−6.9	[−12.8, −1.0]	0.023 *	−7.5	[−13.7, −1.4]	0.018 *

^a^ = Functional Independence Measure; ^b^ = Functional Reach Test; ^c^ = Timed Up ang Go Test; ^d^ = Nine Hole Peg Test; ^e^ = Box and Block Test. * *p* < 0.05.

**Table 4:** Effect of group allocation on post-intervention and follow-up outcomes once adjusted for baseline FIM scores (EG vs. CG; functional outcomes; n = 30).

	Post-Intervention		Follow-Up	
Outcomes	*p* Value	Effect Size (η^2^)	*p* Value	Effect Size (η^2^)
FIM ^a^	<0.001 *	0.461	<0.001 *	0.435
FRT ^b^	0.016 *	0.197	0.014 *	0.203
TUG ^c^	0.663	0.007	0.767	0.003
NHPT ^d^ (right)	0.013 *	0.207	0.005 *	0.253
NHPT ^d^ (left)	0.571	0.012	0.538	0.014
BBT ^e^ (right)	0.126	0.085	0.024 *	0.174
BBT ^e^ (left)	0.835	0.002	0.894	0.001
DASH disability	0.867	0.001	0.958	<0.001
DASH occupation	0.189	0.063	0.152	0.074

^a^ = Functional Independence Measure; ^b^ = Functional Reach Test; ^c^ = Timed Up ang Go Test; ^d^ = Nine Hole Peg Test; ^e^ = Box and Block Test. * *p* < 0.05.

## Data Availability

The data presented in this study are available on request from the corresponding author.

## Supplementary Materials

The following supporting information can be downloaded at: https://www.mdpi.com/article/10.3390/healthcare12080850/s1, Supplementary Material S1: Neuropilates session protocol.
