# Supplementary material for: Effects of Neuropilates on Functional Outcomes in Chronic Stroke: A Randomized Clinical Trial
Source: Healthcare (Basel). 2024 Apr 17;12(8):850. doi: 10.3390/healthcare12080850 (PMC11049962; doi:10.3390/healthcare12080850)
Supplement: Supplementary file 1 [file healthcare-12-00850-s001.zip › healthcare-2842497-supplementary.pdf]

healthcare

MDPI

Supplementary Materials

Table S1. Neuropilates session protocol.

	**First session of the week**	
	**Exercise**	**Repetitions (Time)**
Warm-up	Breathing	20 times (1 minute)
	Arm Circles	10 times per arm (2 minutes)
	The Spine Stretch	20 times (2 minutes)
Main exercise	The One Leg Circle	10 times per leg (4 minutes)
	Break and explanation of the following exercise	(1 minute)
	The Shoulder Bridge	15 times (2 minutes)
	Break and explanation of the following exercise	(1 minute)
	The Hundred	(2 minutes)
Cool-Down	The Saw	10 times per side (3 minutes)
	Cat Stretch	15 times (2 minutes)
	Second session of the week	
	**Exercise**	**Repetitions (Time)**
Warm-up	Breathing	20 times (1 minute)
	Arm Circles	10 times per arm (2 minutes)
	The Spine Stretch	20 times (2 minutes)
Main exercise	The Shoulder Bridge	15 times (2 minutes)
	Break and explanation of the following exercise	(1 minute)
	The Teaser	8 times (4 minutes)
	Break and explanation of the following exercise	(1 minute)
	Push up	8 times (2 minutes)
Cool-Down	Hip Circles	10 times per leg (3 minutes)
	Cat Stretch	15 times (2 minutes)
